# Relationship between the structure, function and endothelial damage, and vascular ageing and the biopsychological situation in adults diagnosed with persistent COVID (BioICOPER study). A research protocol of a cross-sectional study

**DOI:** 10.3389/fphys.2023.1236430

**Published:** 2023-09-12

**Authors:** Leticia Gómez-Sánchez, Olaya Tamayo-Morales, Nuria Suárez-Moreno, Jesus F. Bermejo-Martín, Andrea Domínguez-Martín, José A. Martín-Oterino, José I. Martín-González, David González-Calle, Ángel García-García, Cristina Lugones-Sánchez, Susana González-Sánchez, Raquel Jiménez-Gómez, Luis García-Ortiz, Manuel A. Gómez-Marcos, Elena Navarro-Matías, ICOPER investigators group

**Affiliations:** ^1^ Primary Care Research Unit of Salamanca (APISAL), Salamanca Primary Care Management, Salamanca, Spain; ^2^ Hospital de la Paz de Madrid, Servicio de Urgencias, Madrid, Spain; ^3^ Institute of Biomedical Research of Salamanca (IBSAL), Salamanca, Spain; ^4^ Research Network on Chronicity, Primary Care and Health Promotion (RICAPPS), Salamanca, Spain; ^5^ Castilla and León Health Service–SACYL, Gerencia Regional de Salud, Valladolid, Spain; ^6^ Centro de Investigación Biomédica en Red en Enfermedades Respiratorias (CIBERES), Instituto de Salud Carlos III, Madrid, Spain; ^7^ Hospital Universitario de Salamanca, Internal Medicine Department, Salamanca, Spain; ^8^ Department of Biomedical and Diagnostic Sciences, University of Salamanca, Salamanca, Spain; ^9^ Hospital Universitario de Salamanca, Cardiology Department, Salamanca, Spain; ^10^ Hospital Universitario de Salamanca, Emergency Department, Salamanca, Spain; ^11^ Department of Medicine, University of Salamanca, Salamanca, Spain

**Keywords:** post-acute COVID-19 syndrome (MeSH), ageing (MeSH), vascular stiffness (MeSH), atherosclerosis (MeSH), endothelial damage, exercise (MeSH), healthy lifestyle (MeSH), diet (MeSH)

## Abstract

**Background:** SARS-CoV-2 infection affects the vascular endothelium, which mediates the inflammatory and thrombotic cascade. Moreover, alterations in the endothelium are related to arterial stiffness, which has been established as a marker of cardiovascular disease. The objective of this study is to analyse how the structure, vascular function, vascular ageing and endothelial damage are related to the biopsychological situation in adults diagnosed with persistent COVID and the differences by gender.

**Methods:** This cross-sectional, descriptive, observational study will be carried out in the Primary Care Research Unit of Salamanca (APISAL) and in the BioSepsis laboratory of the University of Salamanca. The sample will be selected from the persistent COVID monographic office at the Internal Medicine Service of the University Hospital of Salamanca, and from the population of subjects diagnosed with persistent COVID in the clinical history of Primary Care. Through consecutive sampling, the study will include 300 individuals diagnosed with persistent COVID who meet the diagnosis criteria established by the WHO, after they sign the informed consent. Endothelial damage biomarkers will be measured using ELLA-SimplePlexTM technology (Biotechne). Their vascular structure and function will be analysed by measuring the carotid intima-media thickness (Sonosite Micromax); the pulse wave and carotid-femoral pulse wave velocity (cfPWV) will be recorded with Sphygmocor System^®^. Cardio Ankle Vascular Index (CAVI), brachial-ankle pulse wave velocity (baPWV) and ankle-brachial index will be analysed with Vasera VS-2000^®^. The integral assessment of the subjects with persistent COVID will be conducted with different scales that evaluate fatigue, sleep, dyspnea, quality of life, attention, nutrition state, and fragility. We will also evaluate their lifestyles (diet, physical activity, smoking habits and alcohol consumption), psychological factors, and cognitive deterioration, which will be gathered through validated questionnaires; moreover, physical activity will be objectively measured using a pedometer for 7 days. Body composition will be measured through impedance using an Inbody 230. Vascular ageing will be calculated with 10 and 90 percentiles of cfPWV and baPWV. Furthermore, we will analyse the presence of vascular injury in the retina, heart, kidneys and brain, as well as cardiovascular risk. Demographic and analytical variables will also be gathered.

**Discussion:** Arterial stiffness reflects the mechanic and functional properties of the arterial wall, showing the changes in arterial pressure, blood flow, and vascular diameter that occur with each heartbeat. SARS-CoV-2 affects the endothelial cells that are infected with this virus, increasing the production of pro-inflammatory cytokines and pro-thrombotic factors, which can cause early vascular ageing and an increase of arterial stiffness. Persistent COVID is a complex heterogeneous disorder that affects the lives of millions of people worldwide. The identifications of potential risk factors to better understand who is at risk of developing persistent COVID is important, since this would enable early and appropriate clinical support. It is unknown whether vascular alterations caused by COVID-19 resolve after acute infection or remain over time, favouring the increase of arterial stiffness and early vascular ageing. Therefore, it is necessary to propose studies that analyse the evolution of persistent COVID in this group of patients, as well as the possible variables that influence it.

**Clinical Trial registration:** ClinicalTrials.gov, identifier NCT05819840

## 1 Introduction

SARS-CoV-2 infection (COVID-19) affects multiple organs and systems, with a large number of clinical manifestations described in the literature (Luo et al., 2022), causing endothelium injuries, local microthrombotic events and an inflammatory state (Lazzaroni et al., 2021; Clemente-Suárez et al., 2022; Gusev et al., 2022; Sbirkov et al., 2022). The severity of this disease is influenced by age and the presence of cardiovascular risk factors such as hypertension, type-2 diabetes mellitus and obesity, which is probably related to vascular alterations that lead to the former two (Harrison et al., 2021), and the association of low-grade inflammation with obesity (Chiappetta et al., 2020). SARS-CoV-2 can infect endothelial cells through the angiotensin converting enzyme 2 (ACE2), downregulating the expression of this enzyme, consequently losing its protective effects, and thus favouring vascular inflammation and the increase of arterial stiffness and organic injury (Monteil et al., 2020; Gnanenthiran et al., 2022).

At the end of spring 2020, when the efforts began to be implemented to control the first wave of cases, it was observed that, in some patients affected by COVID-19, the symptoms persisted weeks after overcoming the acute phase. All this has been subsequently confirmed, observing that, after acute infection, a considerable percentage of individuals continued to present symptoms. This has generated a situation of uncertainty, for both patients and healthcare professionals, since there were neither certainties nor tools to establish a precise diagnosis, as the latter depends, in most cases, on the testimony of the patient about his/her symptoms and, most importantly, on the impact of the symptoms on his/her functioning and quality of life (Abrignani et al., 2022; Raman et al., 2022). Numerous terms have been used to refer to this new entity (Chaichana et al., 2023); the most accepted terms are persistent COVID and long COVID (Lopez-Leon et al., 2021; Mandal et al., 2021; Abrignani et al., 2022; Sugiyama et al., 2022), and a large number of organisations have attempted to provide a validated definition that is accepted by the scientific community. The multiple definitions that have emerged, since the appearance of the first cases, consider it as a multi-organ disease that affects those patients that have suffered from COVID-19, with persistent symptoms and fluctuating frequency, and without an alternative underlying illness that can explain such symptoms (Abrignani et al., 2022; Rubin, 2020; SemFYC: Manifestaciones Persistentes de la Covid-19, 2020; Siegelman, 2020; Severe Acute Respiratory Syndrome, 2021; Shah et al., 2021; Yang et al., 2021; Rodriguez-Sanchez et al., 2022; Centers for Disease Control, 2022; Rodriguez Ledo, 2021).

Currently, the most accepted definition is the one agreed upon by the WHO; this organisation considers persistent COVID as the post-COVID affectation that occurs in individuals with a history of probable or confirmed infection by SARS-CoV-2, which generally continues three months after the appearance of COVID-19 with symptoms that last for at least two months and which cannot be explained by an alternative diagnosis. The most common symptoms are fatigue, difficulty in breathing, and cognitive dysfunction, although other symptoms that usually affect the quality of life can also occur. The symptoms can be de novo, after the initial recovery from an acute episode of COVID-19, or they may persist from the beginning of the disease. They can also fluctuate or there may be relapses over time (Soriano et al., 2022).

Due to the clinical variability and the lack of a shared and specific definition, as well as to the different data gathered, it is very difficult to know the actual prevalence and rate of persistent COVID, which is demonstrated by the great variation among studies (Abrignani et al., 2022; Sugiyama et al., 2022). Systematic reviews and meta-analyses also report great variability in the prevalence of persistent COVID-19, ranging between 30% and 80% of people infected (Lopez-Leon et al., 2021; Chan Sui Ko et al., 2022; Wulf Hanson et al., 2022). Similarly, the cohorts of patients have described different prevalences. Thus, Hossain et al. 2021, at 31 weeks of follow-up, found that the prevalence of prolonged symptoms of COVID was 16.1%. Another study conducted in the Netherlands revealed that one out of eight adults (12.7%) infected by COVID-19 experienced long-term symptoms (Ballering et al., 2022). The United States National Center for Health Statistics reported that one out of five adults who had suffered from COVID-19 was experiencing persistent COVID (Centers for Disease Control and Prevention, 2022). In Spain, it is estimated that at least 10% of people infected present symptoms that can be attributed to this affectation. (Rodriguez Ledo, 2021) Therefore, assuming the most favourable prevalences, persistent COVID still affects millions of people in the world.

Currently, the following are recognised as risk factors of persistent COVID: being a middle-aged woman (Abrignani et al., 2022; Bai et al., 2022; Tsampasian et al., 2023), having been hospitalised for severe symptoms of COVID-19 during the acute phase (Iqbal et al., 2021) and having a worse initial state of health or previous medical comorbidities (Tsampasian et al., 2023), such as depression (Knight et al., 2022), smoking (Hossain et al., 2021), dyslipidemia (Loosen et al., 2022), previous chronic bronchopathy (Loosen et al., 2022), arterial hypertension (Chan Sui Ko et al., 2022; Loosen et al., 2022), diabetes mellitus (Steenblock et al., 2022), autoimmune diseases (Abrignani et al., 2022) and obesity (Abrignani et al., 2022; Loosen et al., 2022). Lower prevalence is reported in people vaccinated against COVID-19 (Azzolini et al., 2022; Tsampasian et al., 2023), showing an inverse relationship with the number of doses received (Azzolini et al., 2022; Kuodi et al., 2022). However, most people with on-going persistent COVID have not experienced changes in their symptoms after vaccination (Watanabe et al., 2023). In the prospective cohort study of Salud de Enfermeras (Nurses’ Health), six healthy lifestyles were considered before the infection, observing that the larger the number of healthy lifestyles prior to the infection, the lower the risk of persistent COVID after the acute infection (Wang et al., 2023).

Persistent COVID is a multi-systemic affectation, presenting in a wide variety of manifestations, where up to 203 symptoms have been identified (Abrignani et al., 2022), including fatigue, post-effort discomfort, dyspnea, chest pain, cognitive deterioration, sleep disorders, anxiety and depression, muscle pain, brain fog, difficulty in concentrating, mental confusion, anosmia/dysgeusia, headache with limitation of the functional capacity, and deterioration of the quality of life (Stein et al., 2022; Romero-Rodríguez et al., 2023; Zeng et al., 2023). According to a recent meta-analysis, people with persistent COVID show a decrease of the initial symptoms (from 92% in the acute phase to 55% at one month of follow-up), followed by stabilisation of approximately 50% for one year. Six or more months after the acute phase, the odds ratio of factors related to the characteristics of the population increased (from 1.62 to 1.82 in women), whereas the value of the odds ratio of the factors related to the acute phase (severe or critical cases and hospitalisation) decreased, and the neuropsychiatric symptoms showed greater prevalence in the long term (approximately 25%) and longer persistence than the physical symptoms (Alkodaymi et al., 2022). Likewise, the psychological consequences of the disease caused by persistent COVID have been reported in different studies (Mandal et al., 2021; Sher, 2021), although the conclusions are not clear, and thus further research is necessary in this respect (Sher, 2021). One of the main characteristics of the disease caused by persistent COVID is the predominance of neurocognitive affectation, which is specifically manifested through problems of attention and memory (78.2% and 72.6% of cases in Spain, respectively) (Rodriguez Ledo, 2021). A relevant number of subjects who have suffered from COVID-19 present post-traumatic stress syndrome, anxiety, depression and insomnia (Mazza et al., 2020). A previous study has related post-traumatic stress syndrome with arterial stiffness (AS) (Tomlinson and Cockcroft, 2011). Moreover, patients with persistent COVID present high levels of inflammatory biomarkers after the initial infection (Lai et al., 2023), and other studies show a dissociation between the symptoms and the objective findings, using standard diagnostic tests (Zota et al., 2022).

The alteration in the structure of the vascular wall evaluated with carotid artery ultrasound and ankle-brachial index (ABI) along with AS have been used as biomarkers to improve the prediction of cardiovascular risk (Visseren et al., 2021). AS presents with a decrease of artery elasticity, and it is a predictor of risk of cardiovascular diseases of similar or greater importance with respect to other traditional cardiovascular risk factors (Visseren et al., 2021). Thus, AS measured in a non-invasive manner, with carotid femoral pulse wave velocity (cfPWV), brachial-ankle pulse wave velocity (baPWV), and cardio-ankle vascular index (CAVI), has shown a positive association with cardiovascular events (Ohkuma et al., 2017; Zhong et al., 2018; Matsushita et al., 2019; Miyoshi et al., 2021; Yasuharu et al., 2021). AS is mainly determined by age, sex and arterial pressure (Laurent et al., 2019; Visseren et al., 2021). In this way, when the central arteries become more rigid, they maintain their duct functions, although they progressively lose their storage properties (Williams et al., 2018a). AS is related to cardiovascular risk factors, lifestyles, inflammatory factors, and psychological factors (Avolio, 2013; Tsai and Hsu, 2021). Therefore, its detection can play an important role in the prevention of cardiovascular diseases, as its alteration appears before the vascular structure is affected (Williams et al., 2018a). Currently, we can measure vascular structure and function in a non-invasive manner. cfPWV, measured with tonometry, is considered the measure of reference (Williams et al., 2018a). cfPWV depends on the arterial pressure at the time of the measurement, and it reflects the AS of the descending aorta, iliac arteries, the first portion of the femoral arteries, the brachiocephalic trunk, and the common carotid artery, without evaluating the ascending aorta, and it is a measure of central AS (Boutouyrie and Vermeersch, 2010). baPWV, measured by oscillometry, is a measure of peripheral AS (Munakata, 2016). CAVI, also evaluated through oscillometry, analyses the stiffness of the aorta (including the ascending aorta) and the iliac, femoral and tibial arteries; it does not depend on the arterial pressure at the time of the measurement, and it is considered a measure of both central and peripheral AS (Shirai et al., 2011; Namba et al., 2019). AS is associated with the appearance of vascular ageing (Nowak et al., 2018; Laurent et al., 2019), reflecting the dissociation between the chronological and biological age of the major arteries, with their alteration preceding the appearance of cardiovascular events (Williams et al., 2018a; Nowak et al., 2018; Laurent et al., 2019). Vascular ageing is used for the early identification of subjects that could benefit from primary prevention. It is described as a gradual process that involves biochemical, enzymatic and cellular processes in the vascular area combined with epigenetic and molecular alterations, and it is considered that it is a fundamental reflection of biological ageing in general, and a determinant of vascular functioning (Mikael et al., 2017; Nowak et al., 2018). During the last decades, epidemiological studies have been conducted to unravel the determining factors of vascular ageing, drawing great interest, as it shows a stronger relationship with morbimortality by cardiovascular diseases than biological ageing (Nowak et al., 2018; Laurent et al., 2019). In this sense, it is known that early vascular ageing (EVA), normal vascular ageing (NVA) and healthy vascular ageing (HVA) are related to the progression of the deleterious characteristics of arterial functioning, which is influenced by the genetic background, the prevalence of classic cardiovascular risk factors, lifestyles and inflammatory factors (Niiranen et al., 2017; Botto et al., 2018; Ji et al., 2018; Nilsson et al., 2018; Nowak et al., 2018; Foscolou et al., 2019). The effect of physical activity on vascular ageing has been analysed in numerous studies, confirming that it plays an important role by mitigating vascular ageing, increasing the availability of nitric oxide (NO) and reducing oxidative stress and vascular wall inflammation (Andersson et al., 2015; Antunes et al., 2016; Jakovljevic, 2018; Zhang et al., 2018; Kucharska-Newton et al., 2019; Gomez-Sanchez et al., 2020). However, some authors suggest that this effect is different between sexes (Seals et al., 2019) and varies depending on the type of physical activity performed (Li et al., 2015). The benefits of the Mediterranean diet (Martinez-Gonzalez et al., 2019; Papadaki et al., 2020; Liu et al., 2021; Morze et al., 2021) and the influence of smoking habits and alcohol consumption on cardiovascular diseases (Kondo et al., 2019; Gonzalez-Sanchez et al., 2020) is widely demonstrated. Lastly, the psychological factors can worsen the inflammatory response or increase the blood levels of cortisol (Shah et al., 2011). Thus, different psychological components such as anxiety, depression and chronic stress can enhance vascular ageing. Nevertheless, the relationship among the determining factors of vascular ageing continues to be an area of interest for research (Gomez-Sanchez et al., 2022). Therefore, maintaining a process of NVA is fundamental for preserving vascular health and delaying the appearance of cardiovascular diseases (Mikael et al., 2017; Nowak et al., 2018). Currently, there is no consensus on a definition of vascular ageing. In this regard, several authors have defined HVA, excluding hypertensive or diabetic subjects from the group of HVA (Niiranen et al., 2017; Ji et al., 2018; Nilsson et al., 2018), and using the percentiles of cfPWV below 10 or 25 from a reference population, or from their own study population, stratified by age groups, and some authors also take into account the values of arterial pressure. Different definitions of EVA have also been published (Van Bortel et al., 2012; Cunha et al., 2015; Cunha et al., 2017; Botto et al., 2018), which are gathered in several studies that consider the highest percentiles of cfPWV for its definition. Various works have employed the values published by Boutouyrie et al. in a European population as reference values (Boutouyrie and Vermeersch, 2010). Our research group has recently published reference values of the different measures of AS for the Spanish adult population without previous cardiovascular disease (Gómez-Sánchez et al., 2020a). Furthermore, we have analysed AS in a general population without cardiovascular disease, measured with several parameters and using different criteria of vascular ageing, analysing their relationship with lifestyles, cardiovascular risk factors, inflammatory factors and psychological factors (Gómez-Sánchez et al., 2020b; Gomez-Sanchez et al., 2020; GómezSánchez et al., 2020). Therefore, we consider that the best definition of vascular ageing must be that which considers the following variables: age, arterial pressure and sex (i.e., the variables with greatest influence on AS), as well as the percentiles of cfPWV of the study population.

During the COVID-19 pandemic, some studies have evaluated the impact of SARS-CoV-2 infection on AS, showing greater AS among the deceased and subjects who presented longer hospitalisation, which may guide toward the prognostic value of such parameter (Schnaubelt et al., 2021). Another study, conducted in a young population, reported lower vascular function and greater AS in the group affected by SARS-CoV-2 at 3-4 weeks, calculated through reactive hyperemia and cfPWV (Ratchford et al., 2021). In this sense, pulse pressure ≥ 60 mmHg upon admission, as a substitute marker of AS, has been associated with an increase of mortality by all causes (Rodilla et al., 2021). A study performed in India, in three groups of patients with acute SARS-CoV-2 infection stratified according to severity, found an association between severity and AS measured with cfPWV; moreover, in seriously ill patients, AS induced by SARS-CoV-2 infection was greater than that reported in control patients with a high load of cardiovascular risk factors (Kumar et al., 2021). Lastly, it has been observed that the data of vascular ageing induced by COVID-19 improve over time, although such parameters of vascular ageing continue to be altered with respect to the reference values obtained in the measurement conducted at 12 months of infection (Zanoli et al., 2022). AS is also associated with the severity and duration of systemic inflammation and low-grade inflammation (Zanoli et al., 2020). SARS-CoV-2 infection presents with multisystemic inflammation and an altered immune response, which could cause deleterious effects on the blood vessels in the short and long term; in turn, this is associated with the severity of the disease and the persistence of symptoms (Zanoli et al., 2017; Dan et al., 2021), which suggests that it can be the connecting link between AS, vascular ageing and persistent COVID. Although the underlying biological mechanisms are still unknown, an abnormal or excessive autoimmune and inflammatory response can play an important role (Carod-Artal, 2021). Furthermore, these late inflammatory, immunometabolic changes produced by SARS-CoV-2 infection and the unresolved defects of immune cells, if maintained, may contribute to the persistence of the symptoms and the development of persistent COVID (Carod-Artal, 2021). Therefore, markers of endothelial damage may play an important role in the development of long COVID, and one of the hypotheses is that the existence of post-COVID endotheliopathy, predominantly microvascular with accompanying low-grade inflammation, could be behind most of the symptoms of this disease (Fogarty et al., 2021; Ahamed and Laurence, 2022). One of the non-invasive methods for studying endothelial function is the quantification of endothelial biomarkers and inflammatory mediators in plasma, the levels of which increase when the endothelium is activated or damaged, allowing valuable information to be obtained both on the status of the endothelium and on the immunological state of the patient. Previous studies have identified biomarkers of a dysregulated response to SARS-CoV-2 infection (Bermejo-Martin et al., 2020). These biomarkers include endothelial function molecules (ICAM-1, VCAM-1, Angiopoietin-2, Endothelin-1), inflammation (IL-6, IL-15, TNF-alpha), immunosuppression (IL-10, PD- L1), virus control/chemotaxis (CXCL10) and interferon response (IFN-gamma). They all participate in key physiological pathways in the pathogenesis of acute COVID-19 and could remain impaired in patients with long COVID. Their quantification in plasma could provide relevant information on the mechanisms of endothelial pathology and immunological dysfunction in these patients, which could improve the diagnosis, monitoring and treatment of this disease.

To sum up, persistent COVID may affect the entire spectrum of COVID-19 patients, from those with a very mild acute disease to those who are seriously ill. The symptoms are heterogeneous, including fatigue, post-effort discomfort, dyspnea, cognitive deterioration, sleep disorders, anxiety and depression, muscle pain, brain fog, anosmia/dysgeusia, headache and limitation of the functional capacity, which affect the quality of life of the patients. The relationship between AS and vascular ageing is not clear. The starting hypothesis of this study states that individuals diagnosed with persistent COVID present greater alteration in the vascular structure and function, as well as worse vascular ageing, compared to the general population without a diagnosis of persistent COVID, since the vascular system of these people is subjected to complex inflammatory deregulation, which has not been studied to date.

Therefore, the main objectives of this study are: 1) to analyse vascular structure, vascular function, biomarkers of endothelial damage and ageing in Spanish adults diagnosed with persistent COVID, determining the differences between sexes; and 2) to analyse the association of the clinical symptoms, lifestyles (physical activity, diet, alcohol consumption and smoking habits), and psychological, cognitive and inflammatory factors with biomarkers of endothelial damage, vascular structure, function and ageing, determining the differences by sexes. Regarding the secondary objectives of this study, we will analyse the possible differences among individuals with persistent COVID according to whether the infection is caused by the Delta mutation or the Omicron mutation. Furthermore, in order to continue advancing in the recognition and visibility of the patients affected by persistent COVID, they will be given a voice through a focal discussion group, where we will delve into the opinions and experiences of the population to facilitate the transfer of the study to the habitual clinical practice.

## 2 Methods and analysis

### 2.1 Study design and context

This will be a cross-sectional, descriptive, observational study in which we will analyse vascular structure, function and ageing and biomarkers of endothelial damage, and their relationship with lifestyles and psychological and inflammatory factors in adults with persistent COVID. The discussion groups will investigate the opinions and experiences of what the affectation by persistent COVID posed to these patients. The Persistent COVID study (GRS 2501/B/22) was registered in ClinicalTrials.gov (registration number: NCT05819840) in April 2023. In order to know if the subjects with persistent Covid have the parameters of structure, of vascular function, as well as the degree of altered vascular aging, we will use as a reference group, the values of these parameters available in the results of the EVA Study in the general population without previous cardiovascular disease (Gómez-Sánchez et al., 2020a), and we will also compare the results with other reference populations (Determinants of pulse wave velocity, 2010; Engelen et al., 2013; Herbert et al., 2014; Cunha et al., 2015). The study will be developed in the Primary Care Research Unit of Salamanca (APISAL) during the year 2023. In this period, we will select the sample and gather the data through questionnaires and explorations, which are described below. This project was approved by the ‘Ethics Committee of Research with Medicines of the health area of Salamanca’ on 27/06/2022 (CEIm reference code: Ref. PI 2022 06 1048). The SPIRIT verification list (Standard Protocol Items: Recommendations for Interventional Trials) (Chan et al., 2013) is available for this protocol (Supplementary Material S1).

### 2.2 Study population

**FIGURE 1:**
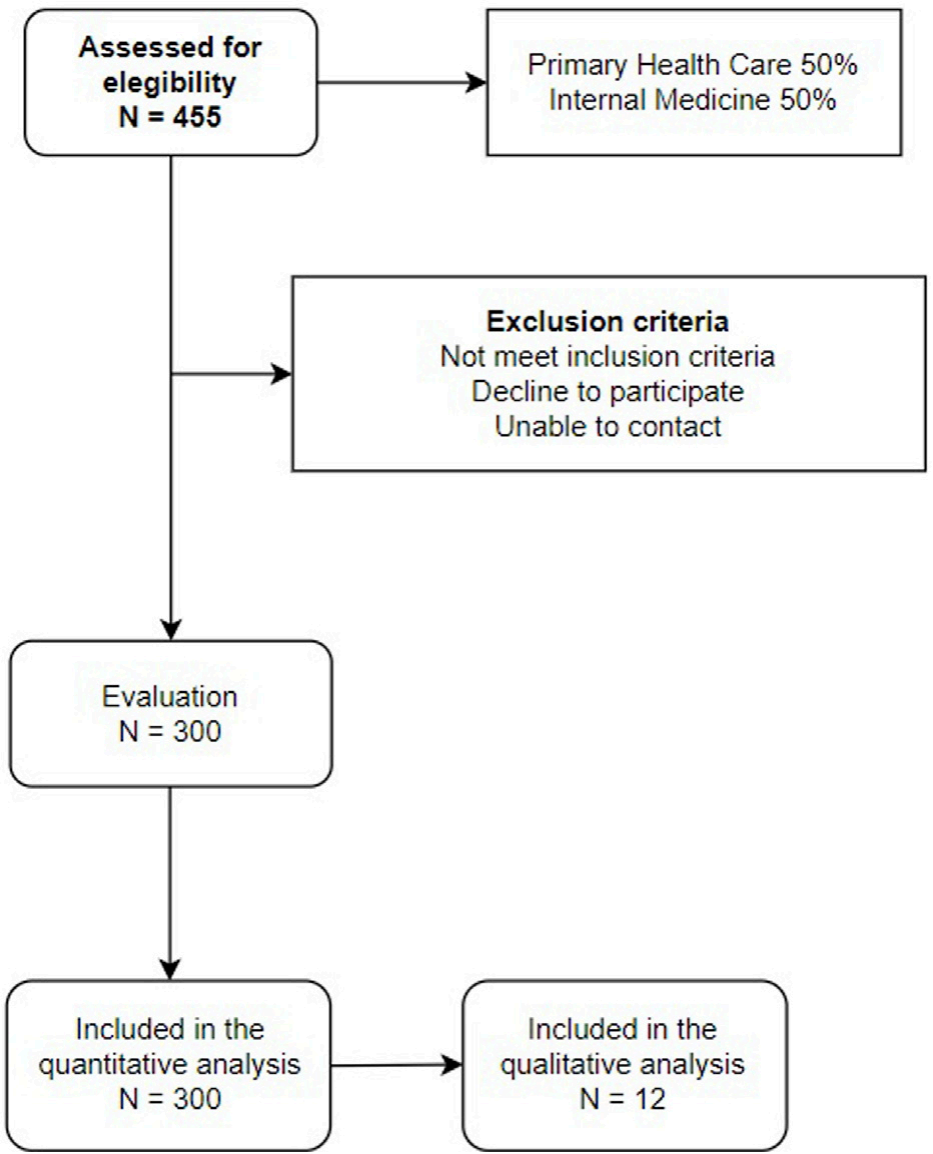
Study flowchart.

Through consecutive sampling, we will select 300 subjects who meet the clinical definition of persistent COVID of the WHO, established by international Delphi consensus, along with patients, their representatives, and experts from the five continents. Persistent COVID is the condition that occurs in individuals with a history of probable or confirmed SARS-CoV-2 infection, generally three months after the onset of the disease, with symptoms that last for at least two months and cannot be explained by an alternative diagnosis. The common symptoms include, among others, fatigue, difficulty in breathing and cognitive dysfunction, and they generally have an impact on daily functioning. They can be de novo after the initial recovery from an acute episode of COVID-19 or persist from the initial disease, and they can also fluctuate or relapse over time (Soriano et al., 2022). The exclusion criteria of the study will be: terminal subjects, subjects who cannot go to the health centres, subjects with a history of cardiovascular disease (ischemic heart disease or cerebrovascular disease) and glomerular filtration rate below 30%. The individuals will be recruited from the registries of the Primary Care offices and the Persistent COVID Office of Internal Medicine in the health area of Salamanca. The sample size was calculated using the GRANMO free software (http://www.imim.cat/ofertadeserveis/software-public/granmo/), detecting a difference in cf-PWV of 0.35 metres/second between subjects with persistent COVID and a sample of Spanish population without cardiovascular disease (Gómez-Sánchez et al., 2020a). Accepting an alpha risk of 0.05 and a beta risk below 0.2 in a bilateral contrast, and assuming a standard deviation of 2.1, a total of 283 individuals are required to detect the indicated difference as significant. Therefore, the 300 subjects that will be included in the study will be sufficient. The subjects included in the study are shown in Figure 1. The patients did not participate in the design of the study; however, they will participate actively in the recruitment, by disseminating the objectives of the study and the inclusion criteria through their organisations. By means of discussion groups, we will investigate the opinions and experiences of the study population about the influence of persistent COVID on vascular health. Similarly, their opinions and attitudes will be considered to facilitate the transfer to the clinical practice. At the end of the study, we will send a detailed report to each patient with the results of the tests conducted. Moreover, a dissemination session will be organised for all the patients included in the study.

### 2.3 Variables and measurement instruments

All measurements will be carried out within a maximum period of 10 days. The three researchers who will perform the measurements will be previously trained, following a standardised protocol, and quality will be controlled by an independent researcher. Table 1 shows the tests applied during the study.

**TABLE 1:** Study variables and measurement instruments.

Sociodemographic variables	
Age, sex, marital status, education level and employment	
Anthropometric variables	
Body weight, height, body mass index, waist circumference, blood pressure	
Body composition	Inbody 230® multifrequency analyzer (Biospace)
Persistent COVID variables	
Fatigue	Modified Fatigue Impact Scale (MFIS)
Sleep quality	Pittsburgh's Sleep Quality Index (PSQI)
Attention disorders	Modified Memory Failures of Everyday (MFE-30)
Dyspnea	Modified Dyspnea Scale (mMRC)
Pain	Spanish chronic pain grade scale
Quality of life	SF-36 health questionnaire
Malnutrition	Malnutrition Universal Screening Tool (MUST)
Fragility	FRAIL scale
Functional evaluation	Functional evaluation scale after COVID-19
Lifestyles	
Diet quality	Mediterranean Diet Adherence Screener (MEDAS)
	Software developed in the Evident study
Physical activity and sedentary time	Global Physical Activity Questionnaire (GPAQ)
	Marshall Sitting Questionnaire (MSQ)
	Digital pedometer (Om ron Hj-321 lay-UPS)
Smoking habits	4-item questionnaire adapted from the MONICA study (WHO)
Alcohol consumption	Questionnaire on alcohol consumption in the last 7 days
Vascular structure	
Carotid intima-media thickness (cIMT)	Sonosite Micromax ultrasound device (FUJIFILM)
Ankle brachial index (ABI)	VaSera VS-2000® device
Vascular function	
Carotid-femoral pulse wave velocity (cfPWV)	SphygmoCor device
Cardio Ankle Vascular Index (CAVI)	VaSera VS-2000® device
Brachial-ankle pulse wave velocity (baPWV)	VaSera VS-2000® device
Cardiovascular Risk	Framingham scale
Target organinjury	
Heart evaluation	ECG (General electric 5000)
Kidney evaluation	Serum creatinine, glomerular filtration rate and albumin-creatinine ratio
Retinal vasculature evaluation	Non-mydriatic retinograph (TOPCON TRC NW 200)
Psychological factors	
Symptoms of anxiety and depression	Goldberg's Anxiety and Depression Scale (GADS)
Cognitive alteration	
Cognitive function	Montreal Cognitive Assessment (MoCA) scale
Endothelial assessment and biomarkers	
Endothelial dysfunction	ICAM-1, VCAM-1, ANGIOPOI ETI N-2, ENDOTHELI N-1
Inflammation	IL-6, IL-15, TNF-ALPHA
Immunosuppression	IL-10, PD-L1
Chemotaxis	CXCL10
Interferon response	I FN-gamm a
Viral load assessment	
Use of digital PCR (dPCR) to quantify viral RNA load in plasma and nasopharyngeal swab	
Correlation with endothelial and immune dysregulation	
Assessment of disease severity	
Analysis of the oresence of SARS-CoV-2 virus reservoirs	

#### 2.3.1 Sociodemographic variables and personal and family background

At the time of inclusion in the study, information about age, sex, marital status, education level and employment will be gathered. We will also gather information about the personal history of hypertension, dyslipidemia, diabetes, hypothyroidism, other diseases and consumption of medicines before the infection and at the time of the study, as well as the consumption of medicines that were required in the acute phase of COVID-19. Furthermore, the family background regarding cardiovascular diseases will also be gathered.

#### 2.3.2 General examination

Height will be measured in cm, with the patient in inhalation, barefoot with the ankles against the wall, using a calibrated wall height rod. With a flexible measuring tape, we will measure the waist perimeter with the tape parallel to the floor, above the iliac crests, at the end of exhalation with the patient standing and in underwear, and the hip perimeter will be recorded at the point of maximum circumference passing by the greater trochanter of both femurs. Body weight and body composition will be determined using an InBody 230 monitor (InBody Co, Ltd, Seoul, South Korea), with the patient fasting for at least 2 hours, barefoot, with light clothes and empty bladder. The body mass index will be calculated as body weight (kg) divided by height squared (m^2^). Clinical arterial pressure will be measured three times, using the mean of the last two measurements, with a validated Omron M10-IT sphygmomanometer (Omron Healthcare, Kyoto, Japan). The measurements will be carried out in the dominant arm of the participant after resting in a sitting position for at least 5 minutes, using a muff of adequate size, which will be determined by measuring the circumference of the upper part of the arm and following the recommendations of the European Society of Hypertension (ESH) (Williams et al., 2018a).

#### 2.3.3 Clinical variables and comprehensive valuation of symptoms of persistent COVID

From the clinical history of the patient, we will gather the vaccination state, the number and date of COVID-19 infections she/he had, whether the diagnosis of the disease was established based on the clinical symptoms or there is confirmation with laboratory tests, and, if so, whether the diagnosis was founded on PCR or Ag test and the variant that caused the acute infection. If the sequencing of the variant is missing, it will be considered that the infection was caused by the predominant variant in the epidemiological week at the time of the infection (data provided by the Territorial Service of Public Health, Epidemiological Service of Salamanca). The symptoms of the acute phase will be recorded through anamnesis. The symptoms presented by the patient due to persistent COVID will be recorded: 1. Systemic manifestations (fatigue, lack of energy, fever); 2. Neurocognitive manifestations (memory loss, difficulty in concentrating, brain fog or confusion); 3. Respiratory/cardiovascular manifestations (dyspnea, weight loss, coughing, sore throat); 4. Musculoskeletal manifestations (muscle pain, joint pain, reduced mobility); 5. Neurological/neuromuscular manifestations (cephalea, taste or smell distorsion, lack of reflexes); and 6. Psychosocial or psychiatric manifestations (depression, anxiety, sleep alterations) (Ceban et al., 2022; Oliveira et al., 2022; Pinzon et al., 2022; Swarnakar et al., 2022; Zeng et al., 2023). We will also obtain information from the patient about the typical symptomatic fluctuation of the disease, asking her/him whether there was a period free of symptoms after the acute infection, whether the symptoms range in intensity, whether she/he presents days free of symptoms, and, if so, how many symptom-free days she/he has had in the last 30 days.

The *comprehensive health valuation* of the subjects affected by persistent COVID will be carried out using the following scales:

The *functional valuation* will be performed with the functional valuation scale after COVID-19 (Severe Acute Respiratory Syndrome, 2021). The questionnaire is divided into two parts (a and b), which will be completed independently. The response options of the two parts will be evaluated from grade 1 to grade 4 as a function of the limitations of the patient.

The *valuation of fatigue* will be conducted using the modified fatigue impact scale (MFIS) (Kos et al., 2005), which consists of 21 questions and evaluates the effects of fatigue during the last 4 weeks, based on a multidimensional approach with cognitive, physical and psychosocial components. Each question is valued from 0 to 4; the total score ranges from 0 to 68, and higher scores indicate greater fatigue.

*Sleep quality* will be evaluated with Pittsburgh’s sleep quality index (PSQI) (Royuela and Macías, 1997), which consists of 10 questions and assesses how the patient’s sleep was during the last month. The first 4 questions refer to specific data about the time the patient goes to bed, the time that it takes him/her to fall asleep, the time when he/she wakes up, and the number of hours he/she sleeps. The following 5 questions reflect more objective data about the sleep problems that the patient suffers from, and the last question is whether the patient sleeps accompanied or alone. The response options are: never in the last month; less than once per week; between once and twice per week; and three or more times per week. The total score may range between 0 and 21 points, with higher scores representing worse sleep quality.

*Attention disorders* will be evaluated using the modified memory failures of everyday (MFE-30) (Montejo et al., 2014). This questionnaire consists of a list of 30 memory failures that may happen in daily living. They are valued as: never or almost never; seldom; sometimes; often; and always or almost always.

*Dyspnea* will be assessed with the modified dyspnea scale (mMRC) (Council, 1966), in a range of 0 to 4, with higher scores indicating greater dyspnea.

*Pain* will be measured using the Spanish chronic pain grade scale (Ferrer-Pena et al., 2016). The questionnaire consists of 8 items about the presence or absence of pain in the patient, its intensity, and its impact on the realisation of activities of daily living, leisure and work in the last three months.

*Quality of life* will be evaluated with the SF-36 health questionnaire (Alonso et al., 1995), which consists of 36 items that gather aspects about physical functioning, physical role, body pain, general health, vitality, social functioning, emotional role and mental health in the last four weeks. Additionally, the SF-36 includes an item about the change in the general health state with respect to the last year.

*Malnutrition* will be assessed using the Malnutrition Universal Screening Tool (MUST) (Scott, 2008; Krznaric et al., 2020). This questionnaire evaluates the BMI, weight loss in the last 3-6 months, and the intake capacity in the last 5 days, classifying the subjects based on their scores in three categories: low risk (0 points), intermediate risk (1 point) and high risk (≥2 points).

*Fragility* will be measured with the FRAIL scale (Morley et al., 2013), which consists of 5 dichotomous questions, valued from 0 to 1, and it analyses five dimensions: fatigue, resistance, aerobic performance, comorbidity and weight loss in the last year. The score ranges from 0 to 5 points, with the following interpretation: probable fragility (3-5 points), probable pre-fragility (1-2 points), and robustness or no fragility (0 points).

#### 2.3.4 Lifestyles

##### 2.3.4.1 Diet quality

*Adherence to the Mediterranean diet* will be measured using the 14-item validated questionnaire Mediterranean Diet Adherence Screener (MEDAS) (Schröder et al., 2011), which was developed by the PREDIMED research group. Each question will be scored as 0 or 1. Adequate adherence to the Mediterranean diet will be assumed for a total score of ≥9 points. With the application developed in the EVIDENT study (Lugones-Sanchez et al., 2022) (registration number: 00/2014/2207), we will record the consumption of food throughout a regular week.

##### 2.3.4.2 Physical activity and sedentary time

*Physical activity* will be objectively evaluated with a validated digital pedometer (Omron Hj-321 lay-UPS) (Steeves et al., 2011), which measures the total steps, the aerobic steps, the distance travelled in Km and the calories burnt in the last 7 days. The participants will carry the pedometer for 9 days, in order to gather the activity performed in 7 full days. These parameters will also be subjectively measured with the global physical activity questionnaire (GPAQ) (OMS, 2014) developed by the WHO. This questionnaire gathers information about participation in physical activity and sedentary behaviour in the last 7 days during work, commuting and leisure time. The doses of physical activity will be estimated in METs/min/week, and those participants who reach 450 METs/min/week will be considered active.

*Sedentary time* will be assessed with the *Marshall Sitting Questionnaire (MSQ)* (Marshall et al., 2010). This questionnaire is validated and evaluates the sitting time in hours and minutes during weekdays and weekends in five domains: commute, work, watching TV, use of computers at home, and unspecified leisure in other domains. The total daily sedentary time will be calculated by adding the session time for each domain.

##### 2.3.4.3 Alcohol consumption and smoking habits

Standardised questionnaires will be used to evaluate the consumption of alcohol and smoking habits. The latter will be assessed through a questionnaire with four standard questions adapted from the MONICA study conducted by the WHO (The World Health Organization MONICA, 1988). The participants of the study will be classified as current smokers or non-smokers (if they have never smoked or have not smoked in the last year). To evaluate the consumption of alcohol, we will employ a structured questionnaire that will assess the intake of alcoholic beverages in the previous week, estimating the grams/week consumed, and classifying the patients in the following categories according to the criteria of the Spanish Health Ministry: teetotaller, low risk, intermediate risk, and risk consumption (Sanidad Md: Límites de Consumo de Bajo Riesgo de Alcohol, 2020).

#### 2.3.5 Vascular structure

Vascular structure will be evaluated using a Sonosite Micromax ultrasound device (FUJIFILM Sonosite, Washington, USA), measuring the carotid intima-media thickness (cIMT), using a high-resolution, 5-10 Mhz multifrequency, lineal transducer and the Sonocal software, which makes automatic measurements of cIMT to optimise the reproducibility (Gómez-Marcos et al., 2012). The carotid ultrasound will be carried out by researchers who will be trained for this purpose before the beginning of the study. Common carotid measurements will be performed after the examination of a 10 mm longitudinal section at 1 cm from the fork. Measurements will be made in the proximal wall and in the distal wall in the lateral, anterior and posterior projection, following an axis perpendicular to the artery in order to discriminate between two lines: one for the lumen-intima interface and the other for the media-adventitia interface. The measurements will be obtained with the patient lying down, head extended and slightly tilted opposite to the examined carotid artery. Pathological thickening of the intima media will be considered for cIMT > 0.9 mm, atheromatous plaque diameter over 1.5 mm, or focal increase of 0.5 mm or 50% of adjacent IMT (Williams et al., 2018b). ABI will be measured using a VaSera VS-2000® device (Fukuda Denshi Co, Ltd, Tokyo, Japan).

#### 2.3.6 Vascular function

Vascular function will be assessed by measuring the carotid-femoral pulse wave velocity (cfPWV), carotid-radial pulse wave velocity (crPWV), the central augmentation index (CAIx) and other hemodynamic parameters, the cardio ankle vascular index (CAVI) and the brachial-ankle pulse wave velocity (baPWV). cfPWV, crPWV and CAIx will be estimated using a SphygmoCor device (AtCor Medical Pty Ltd, central office, West Ryde, Australia). The pulse waves of the carotid, radial and femoral arteries will be analysed, with the patient in the supine position, estimating the delay time with respect to the r wave of the electrocardiogram (ECG) and calculating the cfPWV and crPWV. The distance measurements will be recorded using a measuring tape from the sternal notch to the carotid and femoral arteries and carotid and radial arteries, i.e., where the sensor will be. We will calculate the Arterial Stiffness Gradient Using the ratio of carotid-femoral pulse wave velocity (PWV) to carotid-radial PWV (Fortier and Agharazii, 2016; Pereira et al., 2021). With the patient in a sitting position and leaning his/her arm on a rigid surface, the pulse waves will be analysed with a sensor on the radial artery, using a mathematical transformation to estimate the aortic pulse wave and CAIx (Boutouyrie and Vermeersch, 2010).

CAVI and baPWV will be estimated using a VaSera VS-2000 device (Fukuda Denshi Co, Ltd, Tokyo, Japan), following the manufacturer’s instructions. The values of CAVI will be automatically calculated by replacing the parameters of stiffness in the following equation to detect the vascular elasticity and caPWV: stiffness parameter β=2 ρ × 1/(Ps–Pd) × ln (Ps/Pd) × PWV, where ρ is blood density, Ps and Pd are PAS and PAD in mmHg, respectively, and VOP is measured between the aortic valve and the ankle. The electrodes will be connected to the right and left arms and ankles, and a cardiac sound microphone will be fixed with double-sided adhesive tape to the sternum by the second intercostal space. The participants will remain still and quiet for 5 minutes. Only the CAVI measurements obtained during at least 3 consecutive heartbeats will be considered valid (Shirai et al., 2011). baPWV will be estimated using the following equation: baPWV= (0.5934 × height(cm)+14.4724)/tba, where tba is the time interval between brachial waves and ankle waves (Yamashina et al., 2002; RVfAS, 2010). The CAVI values will be classified as: normal (CAVI<8), borderline (8≤CAVI <9) and abnormal (CAVI≥9) (Shirai et al., 2011; Kawai et al., 2013).

#### 2.3.7 Vascular ageing

Vascular ageing will be evaluated with cfPWV, baPWV or vascular age estimated using a VaSera VS-2000 device (Fukuda Denshi Co, Ltd, Tokyo, Japan) as measures of arterial stiffness. Firstly, the subjects who present vascular injury (injury in the carotid artery or peripheral arteriopathy) will be classified as EVA. Secondly, the subjects will be classified as EVA, NVA and HVA if the values of cfPWV or baPWV are above percentile p90, between p10 and p90, and below p10, respectively. (Gomez-Sanchez et al., 2020)

#### 2.3.8 Determination of biomarkers of endothelial damage

At the Salamanca Primary Care Research Unit (APISAL), 6 mL of blood will be extracted in 2 3K-EDTA tubes, then centrifuged (10 min at 2,500 rpm), and the plasma obtained will be frozen at −20°C. Within a maximum period of 1 month, samples will be transferred in specific freezers for biological samples of an infectious nature which will maintain the correct temperature during transport in dry ice, to the Laboratory for Biomedical Research in Respiratory Infection and Sepsis (BioSepsis) at the Faculty of Medicine at the University of Salamanca, where they will be kept at −80°C. In the BioSepsis laboratory, the plasma concentration of the following biomarkers will be measured using ELLA-SimplePlexTM (Biotechne) technology: ICAM-1, VCAM-1, Angiopoietin-2, Endothelin-1, IL-6, IL -15, TNF-alpha, IL-10, PD-L1, CXCL10, IFN-gamma, according to manufacturer’s specifications. ELLA-SimplePlex technology is a microfluidic-based immunoassay which allows biomarkers to be quantified in 25 ul of plasma in a multiplex format, with extremely high reproducibility and in just 90 min. This system uses cartridges pre-loaded with everything necessary for biomarker quantification, including the calibration curve. To perform the assay, the sample will be diluted in the buffer provided, loaded into the cartridge, and introduced into the ELLA-SimplePlex system. The entire process is then automated: the sample and buffer mixture passes through a microfluidic channel which has antibodies binding the protein of interest. The system then washes off any analyte not bound to the antibody and adds the detection reagent. Because each channel has three nanoreactors coated with the capture antibody, the system provides each result in triplicate. The concentrations of each analyte in pg/ml will be obtained from the calibration curve using the system’s software. This technology allows high reproducibility and will permit results obtained in this project to be compared to those already published by our group in patients during the acute phase of SARS-CoV-2 infection (Bermejo-Martin et al., 2020). The laboratory has reference values in healthy people.

#### 2.3.9 Target organ injury

Kidney and heart evaluation: Kidney function will be assessed through the glomerular filtration rate estimated with the equation of the Chronic Kidney Disease Epidemiology Collaboration (CKD-EPI) (Levey et al., 2009) and the albumin-creatinine ratio, following the ESH criteria (Williams et al., 2018b). The heart will be examined by ECG. Left ventricular hypertrophy will be considered for Sokolow-Lyon index >3.5 mV, or Cornell VDP >2440 mV × ms (Williams et al., 2018b).

Retinal vasculature assessement: Nasal and temporal images focused on the optic disc of both eyes, taken with the subject in a sitting position, will be obtained by a qualified researcher using a TOPCON TRC NW 200 non-mydriatic retinograph (Topcon Europe BC, Capelle aan den IJssel, Netherlands). Employing the ALTAIR software (registration number: 00/2015/995), specifically developed by our research group, we will measure the thickness of the arteries, the thickness of the veins, and the artery/vein index; the area and length of the retina will be calculated semiautomatically (Maderuelo-Fernandez et al., 2020)

#### 2.3.10 Psychological factors and cognitive alteration

The symptoms of anxiety and depression will be evaluated using the Spanish version (Monton et al., 1993) of Goldberg’s Anxiety and Depression Scale (GADS) (Goldberg and Hillier, 1979), which contains a subscale of anxiety (questions 1–9) and a subscale of depression (questions 10–18). The cut-off point is 4 or higher for the anxiety subscale and 2 or higher for the depression subscale. Cognitive function will be evaluated using the Montreal Cognitive Assessment (MoCA) scale (Nasreddine et al., 2005), which is a tool designed for the evaluation of cognitive performance that has been validated in Spain (Lozano et al., 2009). The MoCA assesses the following cognitive domains: attention and concentration, executive functions, memory, language, visuoconstruction abilities, conceptual thinking, calculation and orientation. The maximum score is 30 points, with a score of 26 or higher indicating normal cognitive performance.

#### 2.3.11 Analytical tests

Venous blood samples and urine samples will be collected, between 8 a.m. and 9 a.m., with the participants fasting and avoiding smoking or consuming alcohol or caffeine for 12 h. At the time of inclusion, At the time of inclusion, an analysis will be carried out to determine basal glycemia, urea, uric acid, creatinine, glomerular filtration rate estimated with the CKD-EPI equation, ionogram including calcium, thyroid function, lipid profile, complete blood count measuring (leukocytes, lymphocytes, neutrophils, and eosinophils) iron, ferritin, folic acid, liver profile including Gamma-GT, fibrinogen, haptoglobin, D-dimer, ESR, PCR, and albumin and urine albumin-creatinine ratio. We will also gather, if they exist, the analytical data during the acute episodes. The samples will be collected in the San Juan health centre, where the APISAL research unit is located; then, they will be sent by preferred internal circuit to the Clinical Analyses Service of the University Assistance Complex of Salamanca in collaboration with the Biochemistry and Immunochemistry Unit of CAUSA. The samples will be coded and the analytical techniques used will be those standardised by the laboratory.

### 2.4 Statistical analysis

The data will be registered using the REDCap platform (Research Electronic Data Capture) (Harris et al., 2009; Harris et al., 2019) with a data-gathering questionnaire designed for the project. The normal distribution of the variables will be verified through the Kolmogorov-Smirnov test. The arterial stiffness variables will be standardized and expressed as Z scores to compare results with other reference populations. The analysis of the difference of means between variables of two categories will be performed through Student’s t-test or Mann-Whitney U-test, depending on each case, whereas the qualitative variables will be analysed through X^2^ test. To analyse the relationship between qualitative variables of more than two categories and quantitative variables, an analysis of variance (ANOVA) will be conducted, and a Bonferroni’s test will be performed for the post hoc analyses. The Kruskal-Wallis test will be applied in those cases in which the variables do not show a normal distribution. The analysis of covariance (ANCOVA) will be carried out to control for the variables that can be affected by the results as confounding factors. The relationship among quantitative variables will be analysed using Pearson’s or Spearman’s correlation, depending on each case. In the multivariate analysis, we will use multiple linear regression and logistic regression analyses, with the necessary adjustments according to the type of variables analysed. All variables will be analysed disaggregated by sex, and, whenever applicable, the differences will be analysed from a gender perspective, considering the known influence of gender on many pathologies, particularly cardiovascular and cerebrovascular diseases. The data will be analysed using the SPSS v28.0 statistical package for Windows (IBM, Armonk, New York: IBM Corp). Statistical significance will be set at p < 0.05.

## 3 Discussion

The aim of this study is to analyse the vascular structure, the vascular function and biomarkers of endothelial damage in subjects diagnosed with persistent COVID. Moreover, we will conduct a comprehensive analysis of the health of the individuals affected by persistent COVID, examining their quality of life, fatigue, dyspnea, attention capacity, fragility, sleep, and state of anxiety or depression, using validated scales. We will also investigate the influence of the different lifestyles on the evolution of this disease, as well as the degree of vascular ageing caused by persistent COVID.

Persistent COVID is a new entity, for which different terms and multiple definitions have been used (Lopez-Leon et al., 2021; Mandal et al., 2021; Abrignani et al., 2022; Sugiyama et al., 2022). Due to this variability, the absence of a standardised definition and the lack of gathered data, no reliable prevalence data are available. Similarly, the symptoms are very heterogeneous, affecting the multiple organs and systems (Stein et al., 2022; Romero-Rodríguez et al., 2023; Zeng et al., 2023). Although up to 203 symptoms have been identified (Abrignani et al., 2022), the most common symptoms are: fatigue, dyspnea, chest pain, muscle pain, anxiety, headache with reduced functional capacity, and affectation of the quality of life (Stein et al., 2022; Romero-Rodríguez et al., 2023; Zeng et al., 2023). It has been reported that these symptoms, after the acute phase, decrease during the first month, although then they tend to stabilise and, over time, the physical symptoms improve, but not the neuropsychiatric symptoms, which show greater prevalence in the long term (Alkodaymi et al., 2022). Other authors have also identified risk factors for the development or persistent COVID, such as being a woman, initial severity and comorbidities (Tsampasian et al., 2023), as well as protection factors, such as being vaccinated (Azzolini et al., 2022; Kuodi et al., 2022) and following healthy lifestyles before the acute infection (Wang et al., 2023).

Likewise, Lambadiari et al. (2021) published the results of a study with 70 patients, in which those diagnosed with COVID-19 presented persistent AS and endothelial dysfunction at least 4 months after the initial infection, suggesting a long-term impact on patients with persistent COVID both in AS and in endothelial function (Ikonomidis et al., 2022). However, cfPWV did not vary with the severity of COVID-19, which indicates that vascular dysfunction persists regardless of the initial severity of the disease, although this hypothesis must be confirmed in larger cohorts (Mavraganis et al., 2022; Zota et al., 2022). It is also known that COVID-19 infection invades endothelial cells (endotheliitis) (Varga et al., 2020), and the subsequent endothelial dysfunction is associated with a bad result (Zota et al., 2022). As such, the endothelium plays a double role in the physiopathology of COVID-19: a target organ for the infection and a mediator in the subsequent inflammatory and thrombotic cascades (Mavraganis et al., 2022; Zota et al., 2022). This endothelial dysfunction may extend beyond the acute phase and influence the prognosis of persistent COVID (Lambadiari et al., 2021). Thus, in a study with a 12-month follow-up (Ikonomidis et al., 2022), it was found that the patients with COVID-19 presented persistent AS and endothelial dysfunction. Moreover, we can currently evaluate both central and peripheral AS in a non-invasive manner (Boutouyrie and Vermeersch, 2010; Munakata, 2016; Namba et al., 2019). Therefore, the European Society of Cardiology states that an exhaustive monitoring and further research are necessary to address the possible therapeutic and prognostic implications of endotheliitis induced by COVID-19, recommending an evaluation of AS as a marker of the result of COVID-19 (Evans et al., 2020). In this sense, authors such as Saeed et al. point out that COVID-19 infection and AS have a bidirectional cause-effect association (Saeed and Mancia, 2021). Lastly, it is important to highlight that the number of patients included in the studies that analyse these aspects is small; thus, further research is necessary to determine the impact of persistent COVID on AS and on vascular ageing.

In conclusion, persistent COVID is emerging as an important public health problem. Currently, there are many questions to answer: it is still unclear what the prevalence is, how the symptoms will evolve in the long term, and what the rate will be in people infected after the vaccinations and in those affected by the different variants of SARS-CoV-2 (e.g., Delta, Omicron). Moreover, the literature suggests that COVID-19 causes early vascular ageing and increased AS. However, it is not clear whether this vascular alteration affects the entire vascular tree. Therefore, measuring AS in the whole vascular tree (central and peripheral), which will be carried out in this study, will help to improve our understanding of the vascular affectation of COVID-19, and its prognostic importance will help to characterise it in its entirety, which is an essential step in the management of its evolution. Moreover, it is necessary to perform studies that analyse the reversibility of the vascular changes induced by COVID-19 and their impact on the prognosis in the long term, taking into account that the new and emerging variants could have different effects.

The main limitations of this study should also be highlighted. Firstly, the participants will not represent a random sample of the population diagnosed with persistent COVID. Secondly, due to the cross-sectional nature of the study, it will not be possible to infer causality, although it will allow analysing the associations and generate hypotheses for future prospective studies. On the other hand, the project has strengths that must be underlined, such as the fact that this will be the first study in a Spanish population to analyse the alteration in vascular structure and function, as well as vascular ageing, in patients diagnosed with persistent COVID, exploring the determinants of this disease, as well as the possible role of the inflammatory process in it. In turn, the study will also allow analysing the quality of life of these patients, including the physical, psychological and cognitive aspects, for a better primary care, providing clues for future interventions in the daily care practice. It is also worth mentioning the role of the patients, i.e., those directly affected by persistent COVID, since their role will be key in their visibility and recognition, and they will actively participate in the project through focal discussion groups, in which we will analyse the opinions and experiences of the affected population.

## 4 Brief summary

Persistent COVID is an entity that affects a high percentage of people infected with SARS-CoV-2. This virus can infect the vascular endothelium, which, in turn, causes endothelial alterations that are related to arterial stiffness. Therefore, it is necessary to study the relationship between persistent COVID and the alterations in arterial stiffness. Thus, we propose this study, which is aimed at analysing vascular structure, function and ageing in Spanish adults diagnosed with persistent COVID, determining the differences between sexes. Moreover, it is important to analyse the association of the clinical symptoms, body composition, lifestyles (physical activity, diet, consumption of alcohol and smoking habits), psychological and cognitive factors and inflammatory factors with vascular structure, function and ageing, determining the differences between sexes. The identification of potential risk factors will help to detect people at greater risk of developing persistent COVID, which will enable an early and appropriate clinical support. Furthermore, it is not clear whether the vascular alterations caused by COVID-19 are resolved after the acute infection or remain over time, favouring the increase of arterial stiffness and early vascular ageing. Consequently, it is necessary to propose studies that analyse the evolution of this disease in this group of patients, as well as the possible variables that influence it.

## 5 ICOPER Investigators Group

Carmen Patino Alonso, Alicia Hortega Andrés, Jesús F. Bermejo Martín, David González Calle, Teresa Muñoz Cidad, David Cembrero Fuciños, Ángel García García, Manuel Á. Gómez Marcos, Raquel Jiménez Gómez, José A. Maderuelo Fernández, Andrea Domínguez Martín, Miguel Marcos Martín, José I. Martín González, José A. Martín Oterino, Nadia García Mateo, Elena Navarro Matías, Olaya Tamayo Morales, Nueria Suárez Moreno, Luis García Ortiz, Ana Belén Castro Rivero, Pedro L. Sánchez Fernández, Cristina Lugones Sánchez, Emiliano Rodríguez Sánchez, Leticia Gómez Sánchez and Susana González Sánchez.

## Abbreviations

APISAL, Primary Care Research Unit of Salamanca; cfPWV, carotid-femoral pulse wave velocity; baPWV, brachial-ankle pulse wave velocity; CAVI, cardio-ankle vasculat index; AS, arterial stiffness; EVA, early vascular ageing; NVA, normal vascular ageing; HVA, healthy vascular ageing; CAIx, central augmentation index; MFIS, modified fatigue impact scale; PSQI, Pittsburgh’s sleep quality index; MFE-30, modified memory failures of everyday questionnaire; SF-36, short form-36 health survery; MUST, Malnutrition Universal Screening Tool; FRAIL, Fragility scale; RICAPPS, Network of Research in Chronicity, Primary Care and Health Prevention and Promotion; GPAQ, Global Physical Activity Questionnaire; MEDAS, Mediterranean Diet Adherence Scale; MSQ, Marshall Sitting Questionnaire; cIMT, carotid intima-media thickness; MoCA, Montreal Cognitive Assessment.
